# A rapid molecular assay for the detection of hypervirulent *Klebsiella pneumoniae* in the context of antimicrobial resistance surveillance

**DOI:** 10.1128/spectrum.01068-26

**Published:** 2026-07-27

**Authors:** Valentina Dimartino, Claudia Rotondo, Ivano Petriccione, Marco Favaro, Carla Fontana

**Affiliations:** 1 Laboratory of Microbiology and Biobank, National Institute for Infectious Diseases "Lazzaro Spallanzani"−IRCCS 18653 , Rome, Italy; 2 Department Experimental Medicine, University of Rome Tor Vergata 9318, Rome, Italy; Universita degli Studi dell'Insubria, Varese, Italy

**Keywords:** Hypervirulent *Klebsiella pneumoniae*, real-time PCR, patent protection, whole-genome sequencing, string test, multidrug resistance

## Abstract

Hypervirulent *Klebsiella pneumoniae* (hvKP) represents an emerging clinical and public-health concern, particularly as hypervirulence increasingly converges with multidrug resistance. Current diagnostic approaches rely on phenotypic assays, such as the string test, or on whole-genome sequencing (WGS), both of which have limitations in specificity, turnaround time, standardization, and feasibility for routine surveillance. To address this gap, we developed a multiplex real-time PCR assay targeting key hvKP-associated virulence loci, including siderophore systems, hypermucoviscosity regulators, and additional markers linked to invasive potential. The assay was evaluated on 110 *K. pneumoniae* clinical isolates and 9 positive blood cultures, using WGS and the string test as comparators. The molecular panel demonstrated high concordance with WGS for principal virulence determinants, correctly identifying all high-virulence (score 4) profiles, and most intermediate profiles. Against WGS, the assay yielded a sensitivity of 82% and a specificity of 73%; performance against the string test was 96% and 87%, respectively. Direct testing from blood culture pellets yielded results consistent with both WGS and DNA-based PCR for the limited number of targets detected, supporting the technical feasibility of this approach. However, broader validation is needed to confirm performance in this specimen type. Overall, this multiplex PCR assay provides a targeted molecular screening approach for the rapid identification of hvKP-associated virulence profiles. Its agreement with genomic data supports its potential utility as an accessible complement to WGS for hvKP surveillance, although further workflow optimization will be required before broader routine implementation.

**IMPORTANCE:**

The global emergence of hypervirulent and multidrug-resistant *K. pneumoniae* represents a major public-health threat, as the convergence of virulence and antimicrobial resistance dramatically limits therapeutic options and increases the likelihood of severe, invasive, and potentially untreatable infections. Rapid identification of essential virulence determinants is therefore critical for timely clinical management and for preventing onward transmission. However, current diagnostic approaches are either insufficiently sensitive or require substantial resources, limiting their routine use. By providing a rapid and targeted molecular assay capable of detecting the principal loci associated with hypervirulent *K. pneumoniae* and by demonstrating the preliminary feasibility of its use directly on blood culture pellets previously identified as *Klebsiella* spp. by MALDI-TOF MS, this work provides a pragmatic approach for early virulence profiling. Implementation of such assays can significantly enhance epidemiological surveillance, support tailored patient management, and reduce the spread of high-risk *K. pneumoniae* lineages in both community and healthcare environments.

## INTRODUCTION

Multidrug-resistant (MDR) *Klebsiella pneumoniae* is considered a global public health threat by leading international health organizations (1, 2) due to its rapid spread and high morbidity and mortality rates (3–5). Furthermore, the economic burden associated with its treatment and control is significant (6, 7). In recent decades, high-risk MDR clones (e.g., sequence types [STs] ST512, ST307, ST101) have become endemic in several countries, including Italy (8, 9). At the same time, the spread of hypervirulent *K. pneumoniae* (hvKP) has begun to be documented in immunocompetent individuals (e.g., ST23 and ST86) (10–12) and is increasingly associated with community-acquired infections (13). hvKP is a clinically significant pathotype capable of causing severe pyogenic infections, often with metastatic dissemination. Reported clinical presentations include liver abscess, pneumonia, urinary tract infection, sepsis, meningitis, endophthalmitis, and wound infections (7, 13, 14). The pathogenicity of hvKP is driven by a combination of virulence factors, including a hypermucoviscous phenotype, siderophore-mediated iron acquisition systems (such as aerobactin, yersiniabactin, and salmochelin), and plasmid-encoded regulators, like *rmpA* and *rmpA2*, which enhance capsule production (15, 16).

These features enable hvKP to evade host immune responses and establish infections even in immunocompetent hosts. In fact, the convergence of virulence determinants, with antimicrobial resistance (AMR) markers, including extended-spectrum beta-lactamases and carbapenemases, has been observed (8, 17).

In a recent Italian study, hvKP isolates belonging to ST5, ST8, ST11, and ST25 were found to be the most prevalent and circulating in Italy, although additional STs are now being documented as carriers of virulence genes (18). Although hvKP was initially susceptible to most antibiotics, the emergence of multidrug-resistant hypervirulent strains (MDRhvKP), particularly those carrying carbapenemase genes such as *bla*_KPC_ and *bla*_NDM_, now represents a major therapeutic challenge (19). These strains combine hypervirulence with AMR, severely limiting treatment options and increasing the risk of untreatable infections (16). The European (20, 21) and global (22) dissemination of hvKP, due to the acquisition of plasmids with virulence determinants (23), highlights the need for strengthened surveillance, rapid diagnostic approaches, and novel therapeutic strategies. Currently, hvKP identification relies on two main approaches. The phenotypic identification of hvKP commonly relies on the string test, a simple and cost-effective assay, considered the gold standard for detecting the presence of the mucosal phenotype, which, however, is affected by limited sensitivity and specificity (24), and molecular characterization through whole-genome sequencing (WGS). While this approach is considered the best available method for detecting hypervirulence-associated genes, it is also resource intensive, requiring significant time, financial investment, and specialized expertise. These limitations may contribute to the underestimation of hvKP prevalence among circulating strains in Italy.

In light of the growing link between hypervirulence and AMR in *K. pneumoniae*, the objective of this study was to develop and validate a rapid, cost-effective, real-time PCR (RT-PCR) assay for detecting the key virulence genes associated with the hypervirulent phenotype. Specifically, the study sought to: (i) design a targeted gene panel covering major virulence loci implicated in hvKP pathogenesis; (ii) compare the performance of the RT-PCR assay with WGS; and (iii) assess the feasibility of applying the assay directly to clinical samples, such as blood culture pellets, to support timely surveillance and infection control efforts in healthcare settings.

## RESULTS

### Phenotypic characteristics of the isolates

Antimicrobial susceptibility testing was performed for all 110 isolates, and the corresponding results are shown in Table S1.

### Genotypic characterization of virulence determinants by WGS and real-time PCR

WGS analysis revealed heterogeneous virulence profiles among the 110 *K. pneumoniae* isolates, with virulence scores of 4, 3, 1, and 0 assigned to 27, 7, 67, and 9 isolates, respectively.

Supplementary Table S2 reports the complete strain-by-strain comparison between WGS-derived virulence profiles and RT-PCR findings, together with STs and sequencing coverage, to facilitate interpretation of concordant and discordant results. A complementary summary of WGS-derived resistance genes, WGS virulence scores, and overall RT-PCR virulence profiles is provided in Supplementary Table S3.

For interpretive purposes, isolates carrying the major hypervirulence-associated loci (particularly *iuc*A and/or *peg*-344) were considered to be consistent with a molecular profile associated with an hvKP.

Among the 27 isolates assigned a WGS virulence score of 4, RT-PCR showed broad concordance with the expected hypervirulence-associated profile. *ybtA*, *ybtS*, and *iucA* were detected in all isolates. *rmpA* and *rmpA2* were each detected in 26/27 isolates, whereas *rmpC* and *peg-344* were amplified in 24/27 isolates. *fimH* and *mrkD* were detected in 26/27 isolates. Kleb_04 lacked *rmpC*, *peg-344*, *fimH*, and *mrkD*, while *peg-344* was additionally not detected in Kleb_15 and Kleb_25. Overall, all isolates classified as highly virulent by WGS were recognized by RT-PCR as carrying the principal hypervirulence-associated loci, with only limited target-level discrepancies (Table S3).

Among the seven isolates with a WGS virulence score of 3, RT-PCR consistently detected the *rmp* locus, *iucA*, *fimH*, and *mrkD*. By contrast, detection of yersiniabactin-associated targets was more variable: both *ybtA* and *ybtS* were amplified only in Kleb_27, whereas only *ybtA* was detected in Kleb_29. Notably, Kleb_27 and Kleb_29 were categorized as score 3 by WGS, but their RT-PCR profiles were compatible with a score 4 pattern because they showed the combined detection of major hypervirulence-associated markers. These isolates represented the main cases in which RT-PCR indicated a higher virulence profile than that inferred from WGS-based categorization.

No isolates with a WGS virulence score of 2 were identified in the data set.

Among the 67 isolates with a WGS virulence score of 1, RT-PCR most frequently detected the yersiniabactin-associated targets *ybtA* and *ybtS* together with the adhesion markers *fimH* and *mrkD*, whereas no high-risk hypervirulence-associated combination involving the *rmp* locus and *iucA* was observed in this group. This pattern was consistent with a lower virulence background dominated by yersiniabactin-related determinants rather than by the canonical hypervirulence signature.

Among the nine isolates with a WGS virulence score of 0, RT-PCR did not identify major hypervirulence-associated loci. In this subgroup, only one isolate (Kleb_108) lacked both *fimH* and *mrkD*, while the remaining isolates showed only limited detection of ancillary markers.

Similarly, the nine positive blood cultures displayed very low WGS virulence profiles (scores 0–1), and direct RT-PCR from blood culture pellets detected only a restricted set of virulence-associated genes, mainly *mrkD* and *fimH*, with occasional amplification of *ybtA*/*ybtS* and no evidence of high-risk hypervirulence loci (Table S3).

These findings support the technical feasibility of direct testing from blood culture pellets; however, given the small number of samples and the limited representation of major virulence determinants, they do not allow a formal evaluation of assay performance in blood culture specimens and should therefore be interpreted with caution.

**Fig 1:**
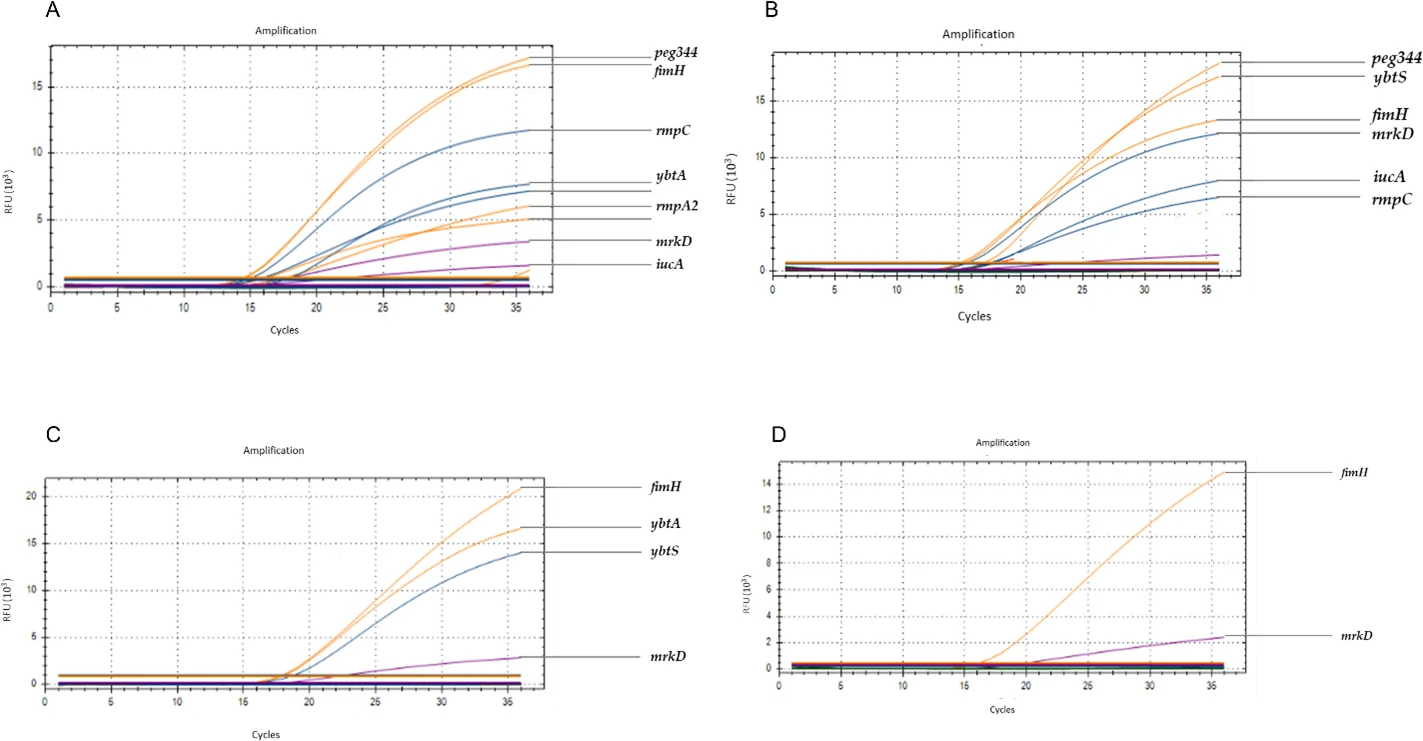
Real-time PCR amplification curves of virulence-associated genes. (**A**) Amplification curves of virulence-related genes in a strain with virulence score 4. (**B**) Amplification curves of virulence-related genes in a strain with virulence score 3. (**C**) Amplification curves of virulence-related genes in a strain with virulence score 1. (**D**) Amplification curves of virulence-related genes in a strain with virulence score 0.

Figure 1 illustrates representative amplification profiles corresponding to isolates with different virulence backgrounds, while Table S2 reports the complete strain-by-strain and target-by-target comparison between WGS-derived virulence profiles and RT-PCR findings, together with ST and sequencing coverage.

### Analytical specificity and cross-reactivity

No amplification was observed for any of the non-target bacterial species included in the exclusivity panel, which confirms the absence of cross-reactivity under the tested conditions (Table S4). Consistent amplification results across all technical and biological replicates confirmed reproducibility, with negligible variability in Ct values between experiments. Although numerical limits of detection (LoD) values were not determined for all targets, the assay consistently detected low-template genomic DNA across serial dilutions, showing amplification dynamics comparable to those reported for similar multiplex virulence-gene assays. The expected bacterial loads in the tested conditions are compatible with the analytical sensitivity of the panels, as suggested.

### Statistical analysis

For the purpose of contingency-table analysis, WGS and RT-PCR results were additionally collapsed into binary categories according to the predefined interpretive framework described above; however, because this simplification does not fully capture the complexity of the virulence profiles, detailed strain-by-strain and target-by-target comparisons are reported in Table S2.

Compared with the string test, the assay showed a sensitivity of 96% and a specificity of 87%, with a negative predictive value (NPV) of 0.99 and a positive predictive value (PPV) of 0.68. When evaluated against WGS, sensitivity was 82% and specificity 73%, with corresponding PPV and NPV values of 0.96 and 0.31, respectively. All summary performance metrics are reported in Table 1.

**TABLE 1:** Summary performance of the RT-PCR assay compared with WGS and the string test^*a*^

	WGS +	WGS −
RT-PCR +	81 (TP)	3 (FP)
RT-PCR −	18 (FN)	8 (TN)
Total		110

	String test +	String test −
RT-PCR +	23 (TP)	11 (FP)
RT-PCR −	1 (FN)	75 (TN)
Total		110

^*a*^ Binary classification was used only for summary performance estimates. Detailed strain-by-strain and target-by-target comparisons are reported in Table S2. TP, true positive; TN, true negative; FP, false positive; FN, false negative; PPV, positive predictive value; NPV, negative predictive value; LR+, positive likelihood ratio; LR−, negative likelihood ratio. For comparison of RT-PCR vs WGS, PPV was 0.96, NPV was 0.31, sensitivity was 82%, and specificity was 73%. For comparison of RT-PCR vs string test, PPV was 0.68, NPV was 0.99, sensitivity was 96%, and specificity was 87%.

The specificity of the assay was 73% (8/11; 95% confidence interval [CI], 43.4–90.3) when compared with WGS and 87% (75/86; 95% CI, 78.5–92.7) when compared with the string test.

Likelihood ratios (LRs) were then calculated. For RT-PCR vs WGS, the LR+ was 3.00 (95% CI, 1.14–7.91), and the LR− was 0.25 (95% CI, 0.144–0.435). For RT-PCR vs the string test, the LR+ was 7.49 (95% CI, 4.29–13.09), and the LR− was 0.0478 (95% CI, 0.0070–0.326).

## DISCUSSION

The growing convergence between hypervirulence and multidrug resistance in *K. pneumoniae* underscores the need for rapid and practical molecular screening tools that can complement existing genotypic and phenotypic frameworks (25, 26). While the string test is simple and inexpensive, it is an imperfect surrogate for the underlying virulence repertoire, and WGS, although comprehensive, remains resource intensive and not universally deployable for high-throughput surveillance (13, 24).

In this study, a multiplex RT-PCR assay targeting key hvKP-associated determinants demonstrated strong agreement with WGS for the principal loci that define virulence profiles. Against WGS, the assay yielded 82% sensitivity and 73% specificity; when compared with the string test, sensitivity was 96% and specificity 87%. Likelihood ratios were consistent with effective screening utility (RT-PCR vs WGS: LR^+^ 3.00 and LR⁻ 0.25; RT-PCR vs string test: LR^+^ 7.49 and LR⁻ 0.0478). These differences across comparators are expected because WGS and the string test interrogate distinct biological dimensions—genotypic potential vs a single phenotypic manifestation—and therefore define “positivity” differently (15, 16, 23). Therefore, the moderate specificity observed in comparison with WGS should be understood in the context of these conceptual differences rather than being considered a direct measure of analytical inaccuracy.

This point is particularly relevant when interpreting agreement with the string test, as some apparent RT-PCR false negatives may instead reflect the limited specificity of the phenotypic comparator rather than true molecular misclassification.

Interpretation of agreement statistics should also account for underlying category prevalence: in our data set, completely negative WGS profiles were under-represented, an asymmetry that can bias classification-based estimates and make overall performance metrics appear lower than a direct reading of the raw results would suggest.

Functionally, these findings support a two-tiered approach in surveillance workflows: multiplex RT-PCR as a rapid first-line genotypic screen aligned to high-risk virulence modules, followed by WGS for comprehensive characterization, genomic-context resolution, and adjudication of discordant or borderline profiles (15, 16). The assay also proved compatible with direct testing from positive blood culture pellets, producing results concordant with both extracted-DNA RT-PCR and WGS, thereby suggesting a streamlined option that can shorten time-to-information without compromising analytical reliability. From an implementation perspective, resource requirements matter: in our setting, the RT-PCR-based approach was substantially less resource intensive than WGS and easier to integrate into routine workflows; relative cost differences should, however, be interpreted as setting dependent.

Overall, the multiplex RT-PCR assay offers a targeted screening approach that is biologically relevant and may complement existing genomic and phenotypic strategies. However, additional workflow simplification is required before it can be implemented on a large scale. By rapidly flagging isolates that harbor genetic determinants linked to invasive potential, it complements genomics and phenotype-based methods and is well suited to strengthen routine surveillance where convergent MDRhvKP lineages are an increasing concern (8, 17).

### Clinical implications

Early recognition of hvKP is clinically relevant because these lineages are associated with invasive disease, metastatic spread, and severe outcomes even in otherwise healthy individuals (7, 16, 23). The multiplex RT-PCR assay provides clinically relevant information by rapidly identifying high-risk virulence modules (e.g., siderophores and hypermucoviscosity regulators), enabling laboratory flagging and timely infection-prevention measures while bridging the gap between simple but imperfect phenotypes and comprehensive but slower genomics (13, 24). Its excellent rule-out capacity against the string test (very low LR⁻) supports early exclusion of hvKP-associated profiles in many routine scenarios, focusing confirmatory resources where they are most needed.

It should be noted that the present assay was specifically designed to detect virulence-associated determinants and does not include antimicrobial resistance targets; references to MDR in this study are therefore intended to provide epidemiological context rather than to imply direct assessment of resistance profiles.

The feasibility of direct testing from blood culture pellets further suggests that rapid molecular profiling may be achievable before sequencing; however, given the limited number of samples analyzed and their low representation of major virulence determinants, these findings should be interpreted with caution and do not allow definitive conclusions on clinical applicability. Further studies are therefore required to validate this approach and to assess its performance in clinically diverse scenarios, including severe infections or outbreak investigations where convergent MDRhvKP strains are suspected or emerging (8, 17). Integration into surveillance frameworks may, in the future, enhance early recognition and containment of high-risk *K. pneumoniae* lineages across both community and healthcare environments.

### Limitations

This work has several limitations. First, it is based on a single-center collection with a modest sample size, which may limit generalizability; multicenter validation in diverse epidemiological settings is warranted. Second, RT-PCR is a targeted genotypic approach: despite high concordance with WGS for principal determinants, it does not resolve genomic context (e.g., plasmid architecture and mobility), which is relevant to transmission and evolution (16, 23).

In addition, some apparently discordant results may reflect incomplete representation of specific loci in WGS assemblies, particularly for plasmid-associated virulence determinants when sequencing coverage is limited, although discordant cases in the present data set were not restricted to the lowest-coverage isolates.

Third, although direct testing from positive blood culture pellets yielded results consistent with extracted-DNA RT-PCR and WGS, broader standardization (pre-analytical handling, internal controls, and acceptance criteria) and inter-laboratory reproducibility assessments are needed before routine adoption.

Moreover, the evaluation of the assay on positive blood culture pellets was limited by the small number of samples and by the low prevalence of major virulence determinants in this subset. Therefore, these results should be interpreted as preliminary feasibility data and do not allow definitive conclusions on assay performance in blood culture specimens.

Fourth, the asymmetric distribution of virulence categories (under-representation of fully negative WGS profiles) can bias classification-based performance metrics (e.g., specificity and PPV/NPV) and should be considered when comparing accuracy across non-equivalent reference methods (WGS vs string test). Fifth, while reproducibility and exclusivity were evaluated, numerical limits of detection were not established for all targets, constraining comparative analytic-sensitivity claims. Finally, the study did not include independent phenotypic or *in vivo* measures of virulence or systematic clinical-outcome correlations, limiting inferences on clinical severity attributable to specific gene signatures (7). Future multicenter studies integrating genomic, molecular, phenotypic, and clinical data will be key to harmonize analytical parameters and clarify the prognostic value of defined virulence signatures within MDR–hvKP surveillance strategies.

## MATERIALS AND METHODS

### Hospital setting and capability

The National Institute for Infectious Diseases “Lazzaro Spallanzani” (INMI) is a specialist infectious diseases and AMR center with 160 beds. It provides specialized clinical care, supports surveillance and prevention initiatives, and drives innovation. It is also designated as a reference laboratory for the National Action Plan to Combat AMR. The INMI laboratory has advanced capabilities for typing and characterizing clinically and epidemiologically relevant pathogens, with a focus on AMR determinants and virulence factors. A dedicated biobank supports the long-term preservation of biological samples and microbial isolates for surveillance and research activities.

### Collection of clinical isolates

A total of 110 *K. pneumoniae* isolates (Kp-1 to Kp-110) were included in the study. Isolates were obtained from clinical specimens comprising 63 rectal swabs, 34 urine samples, 7 blood cultures, 4 wound swabs, 1 bronchoalveolar lavage, and 1 nasal swab, all collected and stored in the INMI Biobank. In addition, nine positive blood cultures were included.

### Phenotypic characterization of isolates

Antimicrobial susceptibility testing was performed using the Phoenix system (Becton Dickinson Diagnostics, San Jose, CA, USA), while species identification was carried out using the MALDI-TOF Biotyper Sirius system (Bruker Daltonics, Bremen, Germany). Minimum inhibitory concentration results were interpreted according to the most recent European Committee on Antimicrobial Susceptibility Testing guidelines (27).

The hypermucoviscous phenotype was evaluated using the string test; isolates producing a viscous string ≥5 mm in length when stretched with an inoculation loop were considered positive (28). However, this assay was used exclusively as a preliminary phenotypic screening tool to support the selection of isolates for further analysis. Consistent with previous reports, the string test has limited specificity and does not reliably distinguish hypervirulent from non-hypervirulent strains, as hypermucoviscosity and hypervirulence are related but not fully overlapping phenotypes (14, 29, 30). Therefore, the test was not considered a definitive criterion for hypervirulence, but rather an initial, non-specific indicator of a hypermucoviscous phenotype.

### Real-time PCR design

Following a comprehensive and systematic review of PubMed-indexed literature, we selected a panel of genes consistently associated with hypervirulence in *K. pneumoniae*. The inclusion of each target was not arbitrary, but based on converging evidence supporting its involvement in key virulence pathways, including capsule regulation, iron acquisition, toxin production, and host colonization. This selection strategy was further informed by established genomic frameworks for virulence assessment, particularly the work of Lam et al., which defines hypervirulence based on the accumulation of specific virulence loci rather than single markers (15).

The selected targets included *rmpA*, *rmpA2*, *rmpC*, *rmpD*, *magA*, *cnf-1*, *ybtA*, *ybtS*, *iucA*, *iroB*, *iroN*, *clbA*, *peg-344*, *hlyA*, *fimH*, and *mrkD*. Nucleotide sequences were retrieved in FASTA format from the National Center for Biotechnology Information (NCBI) database, and primers and probes were designed using Primer-BLAST (https://www.ncbi.nlm.nih.gov/tools/primer-blast/index.cgi?LINK_LOC=BlastHome, accessed on 16 March 2026).

All primer and probe sequences were designed and optimized in-house and evaluated for specificity using BLAST-based comparisons against publicly available genomic and patent databases.

The sequences and corresponding oligonucleotides are protected under national (No. 102024000020623) and international (PCT/IT2025/050213) patents.

Due to the fluorophore detection limits of the CFX platform, which restrict the number of fluorescent signals that can be monitored simultaneously, the targets were divided into six multiplex panels. Panels 1 and 2 comprised genes associated with hypermucoviscosity, while panels 3, 4, 5, and 6 included genes linked to hypervirulence.

Preliminary optimization experiments were performed to assess potential interference among primer/probe sets within each multiplex panel, and no significant reduction in amplification efficiency or signal discrimination was observed during assay optimization. Comparable Ct values and amplification profiles were obtained, supporting the analytical robustness of the multiplex design.

Probes were labeled with fluorophores compatible with the CFX optical system (FAM, HEX, ROX, and Cy5) and carefully assigned to minimize spectral overlap while maintaining efficient amplification and clear signal discrimination. Importantly, fluorophore assignment reflected functionally coherent groups of virulence determinants: highly discriminative markers such as *iucA* and *peg-344* were analyzed independently, while genes with overlapping biological roles (e.g., capsule regulators or siderophore systems) were grouped within the same detection channel.

This function-oriented design enabled the direct integration of RT-PCR results into a semi-quantitative virulence scoring system, structured around biologically defined gene clusters. Specifically, the presence of key markers (*iucA* and/or *peg-344*) contributed the highest weight to the score, while additional groups (capsule-associated genes, siderophore systems, toxin-related genes, and adhesion factors) incrementally contributed to the overall score. In this way, each fluorescence signal corresponds to a defined virulence pathway, allowing a transparent and reproducible estimation of virulence potential that reflects the cumulative genetic background rather than isolated gene detection.

Finally, the assay was designed as a targeted molecular screening tool to provide clinically relevant virulence profiling. However, it should be noted that the current multiplex configuration still requires further optimization for broader routine implementation.

### Virulence score calculation

To provide a structured interpretation of the multiplex RT-PCR results, a semi-quantitative virulence scoring system was developed based on the presence of functionally grouped virulence determinants. In this study, hvKP was operationally defined on the basis of the detection of key hypervirulence-associated loci, primarily *iucA* and/or *peg-344*. These were then interpreted in the context of the overall multiplex RT-PCR virulence profile. The RT-PCR-based virulence score was used as a complementary interpretive framework to summarize the cumulative virulence background rather than as a standalone biological definition of hvKP. Consequently, isolates carrying major hypervirulence-associated markers were considered to be consistent with an hvKP-associated profile, whereas lower scores reflected intermediate or low virulence backgrounds.

The scoring framework was designed to reflect the cumulative contribution of key virulence pathways rather than the presence of individual genes, in line with previously proposed genomic approaches for *K. pneumoniae* virulence assessment (15).

Detected targets were categorized into biologically relevant groups, including: (i) key hypervirulence-associated markers (*iucA* and *peg-344*), (ii) capsule regulation and hypermucoviscosity-associated genes (*rmpA*, *rmpA2*, *rmpC*, *rmpD*, and *magA*), (iii) siderophore systems (*iroB*, *iroN*, *ybtA*, and *ybtS*), (iv) toxin-associated genes (*clbA*, *cnf-1*, and *hlyA*), and (v) adhesion-related genes (*fimH* and *mrkD*).

Each functional group contributed to the overall virulence score according to its relative association with hypervirulence. The presence of key markers (*iucA* and/or *peg-344*) was assigned the highest weight (2 points), given their strong and consistent association with hypervirulent phenotypes. Additional functional groups, including capsule-associated genes, siderophore systems, and toxin-related determinants, contributed 1 point each when at least one gene within the group was detected. Adhesion-related genes were considered ancillary contributors and assigned a lower weight (0.5 points), reflecting their broader distribution among both hypervirulent and non-hypervirulent strains.

The total virulence score was calculated as the sum of all detected functional groups, resulting in a semi-quantitative estimate of virulence potential. Based on the distribution of scores, isolates were categorized into four groups: high virulence potential (score ≥3), possible hypervirulent profile (score 2–2.5), intermediate virulence (score 1–1.5), and low virulence potential (score ≤0.5).

This scoring system was not intended as a definitive diagnostic classification of hvKP but rather as a pragmatic and biologically informed tool to facilitate comparison between isolates and to provide an integrated assessment of virulence potential. By aggregating functionally related determinants, the approach reduces the impact of individual gene variability and improves interpretability in the context of a multiplex RT-PCR assay.

In practical terms, RT-PCR results were interpreted according to the presence and combination of major hypervirulence-associated targets, with highly discriminative markers such as *iucA* and *peg-344* carrying greater interpretive weight, whereas more broadly distributed loci such as *fimH* and *mrkD* were considered supportive markers only.

The interpretive framework used to derive and read the RT-PCR-based virulence score is summarized in Tables 2 and 3.

**TABLE 2:** Assignment of the RT-PCR-based virulence score according to functional groups of virulence determinants^*a*^

Functional group	Markers included	Assigned score	Interpretive role
Key hvKP-associated markers	*iucA* and/or *peg-344*	2 points	Highest interpretive weight
Capsule/hypermucoviscosity	*rmpA*, *rmpA2*, *rmpC*, *rmpD*, and *magA*	1 point	Additional virulence-associated background
Accessory siderophores	*iroB*, *iroN*, *ybtA*, and *ybtS*	1 point	Additional virulence-associated background
Toxins	*clbA*, *cnf-1*, and *hlyA*	1 point	Additional virulence-associated background
Adhesion	*fimH* and *mrkD*	0.5 points	Supportive markers only

^*a*^ The RT-PCR-based virulence score was structured around biologically relevant functional groups of virulence determinants. Key markers associated with hypervirulence (*iucA* and/or *peg-344*) were assigned the highest weight, whereas adhesion-related genes were considered supportive markers only.

**TABLE 3:** Interpretation of the RT-PCR-based virulence score^a^

Total score	Interpretation
≥3	hvKP highly likely
2–2.5	hvKP possible
1–1.5	Intermediate virulence profile
0–0.5	Low virulence profile

^*a*^ The virulence score was intended as a pragmatic interpretive tool for RT-PCR-based virulence profiling and not as a definitive diagnostic classification of hvKP. Scores were used to summarize the cumulative virulence background detected by the assay and to help identify hvKP-associated profiles.

### Sample preparation for the molecular assay

Primers and probes were evaluated using DNA extracted from all 110 bacterial isolates with the QIAamp DNA Mini Kit (Qiagen, Hilden, Germany). In addition, the assay was tested directly on nine positive blood cultures, previously identified as *Klebsiella* spp. by MALDI-TOF MS. For each blood culture, a two-step centrifugation protocol was applied to obtain the pellet: samples were first centrifuged at 500 × *g*, after which the supernatant was collected and centrifuged at 5,000 × *g*. The resulting pellet was resuspended in nuclease-free water (Thermo Fisher Scientific Invitrogen).

Amplification of target regions was performed using the qPCRBIO Probe Mix No-ROX (PCR Biosystems Ltd., London, UK) on a CFX96 RT-PCR system (Bio-Rad Laboratories, Hercules, CA, USA).

### Analytical specificity and cross-reactivity

Analytical specificity was assessed using a two-step *in silico* workflow. First, all primer–probe sets and their corresponding target regions were screened using Primer-BLAST against the NCBI database to exclude predicted non-specific annealing and to identify potential cross-species homologies.

Clustal Omega alignments (https://www.ebi.ac.uk/jdispatcher/msa/clustalo; accessed on 26 March 2026) were then used to compare the target region within *Klebsiella* sequences only. This allowed us to identify potential nucleotide differences and assess variability within the amplified locus of the species.

Exclusivity testing in the laboratory was conducted using genomic DNA from non-target bacterial species, applying the same RT-PCR conditions as for clinical isolates. A reaction was classified as non-specific if a sigmoidal amplification curve appeared before the predefined Ct threshold (e.g., Ct < 40) in the presence of a non-target template. Each run included no-template controls and positive controls consisting of DNA from WGS-confirmed clinical isolates, to monitor contamination and amplification performance. A panel of non-target microorganisms commonly detected in the same clinical matrices was selected for exclusivity testing. These included non-fermenters (*Pseudomonas aeruginosa* and *Acinetobacter baumannii*) and gram-positive organisms (*Staphylococcus aureus* and *Enterococcus faecalis*), which were tested at high template input (10⁵–10⁶ genome copies per reaction; Table S4). The reproducibility of the assay was evaluated through multiple technical and biological replicates generated across independent experiments.

The analytical sensitivity of the assay was preliminarily assessed by testing serial dilutions of genomic DNA. However, numerical LoD values were not formally established for all targets.

### WGS and virulence profiling

DNA samples analyzed by RT-PCR were also subjected to WGS using the Illumina MiSeq platform (Illumina, Inc., San Diego, California, USA). All the raw sequencing reads generated were uploaded to the PathogenWatch platform (version 1.1) for genomic analysis of virulence and resistance genes (available at https://pathogen.watch/en, accessed on 16 March 2026). PathogenWatch can identify five key virulence loci: the siderophores yersiniabactin (*ybt*), aerobactin (*iuc*), and salmochelin (*iro*), the genotoxin colibactin (*clb*), and the hypermucoidy locus *rmpADC*. It can also automatically detect acquired AMR genes and relevant chromosomal mutations (29). Virulence scores were obtained using the KLEBORATE online tool (version 3.0; https://github.com/klebgenomics/Kleborate, accessed on 11 February 2026), which assigns a categorical score from 0 to 5 and enables the monitoring of convergence between AMR and virulence traits (25).

For each isolate, the WGS-derived virulence profile, ST, and sequencing coverage were recorded and used for direct strain-by-strain comparison with the corresponding RT-PCR results, as reported in Table S2. Genome coverage was estimated as the ratio between the total number of sequenced bases and the assumed *K. pneumoniae* genome size (5.5 Mb) and is reported as *x*-fold coverage. Sequencing coverage was included to support interpretation of discordant findings, particularly for loci that may be underrepresented in assemblies because of lower plasmid-associated read depth. A complementary summary of WGS-derived resistance genes, virulence scores, and corresponding RT-PCR virulence profiles is reported in Table S3.

### Statistical analysis

The coherence of the results was used to evaluate the performance of the RT-PCR assay, with two reference standards being employed: WGS and the string test. Contingency tables were generated for each comparison, and the following were calculated: sensitivity, specificity, PPV, and NPV.

## Data Availability

The data supporting the findings of this study are available from the corresponding author upon reasonable request. All the raw reads were deposited in the Sequence Read Archive under BioProject ID PRJNA1193862.

## SUPPLEMENTAL MATERIAL

Table S1 spectrum.01068-26-s0001.docx Antimicrobial profiles of the isolates.

Table S2 spectrum.01068-26-s0002.docx Strain-by-strain comparison of WGS-derived virulence profiles and real-time PCR findings in 110 *Klebsiella pneumoniae* isolates, including sequence type and sequencing coverage.

Table S3 spectrum.01068-26-s0003.docx Resistance genes identified through WGS analysis and corresponding virulence scores.

Table S4 spectrum.01068-26-s0004.xlsx Cross-reactivity test results.

ASM does not own the copyrights to Supplemental Material that may be linked to, or accessed through, an article. The authors have granted ASM a non-exclusive, world-wide license to publish the Supplemental Material files. Please contact the corresponding author directly for reuse.
