# Supplementary material for: A rapid molecular assay for the detection of hypervirulent Klebsiella pneumoniae in the context of antimicrobial resistance surveillance
Source: Microbiol Spectr. 2026 Jul 27;14(9):e01068-26. doi: 10.1128/spectrum.01068-26 (PMC13532228; doi:10.1128/spectrum.01068-26)
Supplement: Supplementary file 1 [file spectrum.01068-26-s0001.docx]

|  |  | **MIC (mg/L)** | | | | | | | | |
| --- | --- | --- | --- | --- | --- | --- | --- | --- | --- | --- |
| **Strain** | **Sample** | **CZA** | **ERT** | **MEM** | **IMI** | **GENT** | **AMK** | **CIP** | **TZP** | **COL** |
| Kleb_01 | Urine | >8/4, R | >1 R | >16 R | >8 R | >4 R | >16 R | >1 R | >64/4 R | 0.5 S |
| Kleb_02 | Urine | >8/4 R | >1 R | >16 R | >8 R | >32 R | >32 R | >1 R | >64/4 R | 0.5 S |
| Kleb_03 | Rectal swab | >8/4 R | >1 R | 64 R | >8 R | >8 R | >16 R | >1 R | >64/4 R | >4 R |
| Kleb_04 | Urine | >8/4 R | >1 R | >16 R | >8 R | ≥16 R | >16 R | >1 R | >64/4 R | 2 S |
| Kleb_05 | Urine | >8/4 R | >1 R | >8 R | >8 R | >4 R | >16 R | >1 R | >16/4 R | ≤1 S |
| Kleb_06 | Blood culture | >8/4 R | >1 R | >16 R | >8 R | ≥16 R | >32 R | >1 R | >64/4 R | ≥8 R |
| Kleb_07 | Rectal swab | >8/4 R | >1 R | >16 R | ≥16 R | ≥16 R | >16 R | >1 R | >64/4 R | 0.5 S |
| Kleb_08 | Urine | >8/4 R | >1 R | 64 R | ≥16 R | >8 R | >16 R | >1 R | >64/4 R | 16 R |
| Kleb_09 | Urine | >8/4 R | >1 R | >8 R | >8 R | >4 R | >16 R | >1 R | >16/4 R | ≤1 S |
| Kleb_10 | Rectal swab | >8/4 R | >1 R | >16 R | >8 R | ≤1 S | >16 R | >1 R | >64/4 R | 0.5 S |
| Kleb_11 | Urine | >8/4 R | >1 R | >16 R | >8 R | ≤1 S | >16 R | >1 R | >64/4 R | 0.5 S |
| Kleb_12 | BAL | >8/4 R | >1 R | >16 R | >8 R | ≥16 R | >16 R | >1 R | >64/4 R | 0.5 S |
| Kleb_13 | Rectal swab | >8/4 R | >1 R | >16 R | >8 R | ≥16 R | >32 R | >1 R | >64/4 R | 0.5 S |
| Kleb_14 | Rectal swab | >8/4 R | >1 R | >16 R | >8 R | >4 R | >16 R | >1 R | >64/4 R | 0.5 S |
| Kleb_15 | Nasal swab | >8/4 R | >1 R | >8 R | >8 R | >4 R | >16 R | >1 R | >16/4 R | ≤1 S |
| Kleb_16 | Urine | >8/4 R | >1 R | >8 R | >8 R | >4 R | >8 R | >1 R | >16/4 R | ≤1 S |
| Kleb_17 | Rectal swab | >8/4 R | >1 R | >8 R | >8 R | >4 R | >8 R | >1 R | >16/4 R | >4 R |
| Kleb_18 | Rectal swab | >8/4 R | >1 R | >8 R | >8 R | >4 R | >16 R | >1 R | >16/4 R | ≤1 S |
| Kleb_19 | Rectal swab | >8/4 R | >1 R | >16 R | >8 R | >4 R | >16 R | >1 R | >64/4 R | 0.5 S |
| Kleb_20 | Rectal swab | >8/4 R | >1 R | >8 R | >8 R | >4 R | >16 R | >1 R | >16/4 R | >4 R |
| Kleb_21 | Rectal swab | >8/4 R | >1 R | >16 R | >8 R | 2 S | >16 R | >1 R | >64/4 R | >4 R |
| Kleb_22 | Rectal swab | >8/4 R | >1 R | >16 R | >8 R | 2 S | >16 R | >1 R | >64/4 R | >4 R |
| Kleb_23 | Rectal swab | 256/4 R | >1 R | >8 R | >8 R | >4 R | >16 R | >1 R | >16/4 R | ≤1 S |
| Kleb_24 | Urine | 256/4 R | >1 R | >16 R | ≥16 R | ≥16 R | >16 R | >1 R | >16/4 R | 0.5 S |
| Kleb_25 | Rectal swab | 256/4 R | >1 R | >16 R | >8 R | ≥16 R | >32 R | >1 R | >64/4 R | >4 R |
| Kleb_26 | Rectal swab | 256/4 R | >1 R | >16 R | >8 R | ≥16 R | >32 R | >1 R | >64/4 R | 0.5 S |
| Kleb_27 | Rectal swab | 256/4 R | >1 R | >16 R | >8 R | ≥16 R | >32 R | >1 R | >64/4 R | 0.5 S |
| Kleb_28 | Rectal swab | 256/4 R | >1 R | >16 R | >8 R | ≥16 R | >32 R | >1 R | >64/4 R | 0.5 S |
| Kleb_29 | Blood culture | 256/4 R | >1 R | >16 R | >8 R | ≥16 R | >32 R | >1 R | >64/4 R | 0.5 S |
| Kleb_30 | Rectal swab | 256/4 R | >1 R | >16 R | >8 R | ≤1 S | >32 R | >1 R | >64/4 R | 0.5 S |
| Kleb_31 | Rectal swab | 256/4 R | >1 R | >16 R | >8 R | ≤1 S | >32 R | >1 R | >64/4 R | 0.5 S |
| Kleb_32 | Rectal swab | 256/4 R | >1 R | >16 R | >8 R | ≥16 R | >32 R | >1 R | >64/4 R | 0.5 S |
| Kleb_33 | Rectal swab | 256/4 R | >1 R | >16 R | >8 R | ≥16 R | >32 R | >1 R | >16/4 R | 0.5 S |
| Kleb_34 | Urine | >8/4 R | >1 R | >16 R | ≥16 R | 2 S | >32 R | >1 R | >64/4 R | 0.5 S |
| Kleb_35 | Rectal swab | 256/4 R | >1 R | >16 R | >8 R | ≥16 R | >32 R | >1 R | >16/4 R | 0.5 S |
| Kleb_36 | Rectal swab | 256/4 R | >1 R | 16 R | >8 R | ≥16 R | >16 R | >1 R | >16/4 R | 0.5 S |
| Kleb_37 | Rectal swab | >8/4 R | >1 R | >8 R | >8 R | >4 R | >16 R | >1 R | >16/4 R | ≤1 S |
| Kleb_38 | Urine | >8/4 R | >1 R | >8 R | >8 R | 2 S | >8 R | >1 R | >16/4 R | ≤1 S |
| Kleb_39 | Rectal swab | >8/4 R | >1 R | >8 R | >8 R | >4 R | >16 R | >1 R | >16/4 R | ≤1 S |
| Kleb_40 | Rectal swab | >8/4 R | >1 R | >8 R | >8 R | >4 R | >16 R | >1 R | >16/4 R | ≤1 S |
| Kleb_41 | Rectal swab | >8/4 R | >1 R | >8 R | >8 R | >4 R | >16 R | >1 R | >16/4 R | ≤1 S |
| Kleb_42 | Urine | >8/4 R | >1 R | >8 R | >8 R | 2 S | >16 R | >1 R | >16/4 R | ≤1 S |
| Kleb_43 | Rectal swab | >8/4 R | >1 R | >8 R | >8 R | >4 R | >8 R | >1 R | >16/4 R | ≤1 S |
| Kleb_44 | Stool | 256/4 R | >1 R | >8 R | >8 R | >4 R | >16 R | >1 R | >16/4 R | ≤1 S |
| Kleb_45 | Rectal swab | >8/4 R | >1 R | >16 R | >8 R | >4 R | >16 R | >1 R | >64/4 R | ≤1 S |
| Kleb_46 | Rectal swab | >8/4 R | >1 R | 32 R | >8 R | ≤1 S | >8 R | >1 R | >64/4 R | 0.5 S |
| Kleb_47 | Rectal swab | >8/4 R | >1 R | 32 R | >8 R | ≤1 S | >16 R | >1 R | >64/4 R | 0.5 S |
| Kleb_48 | Rectal swab | 256/4 R | >1 R | >8 R | >8 R | >4 R | >8 R | >1 R | >16/4 R | ≤1 S |
| Kleb_49 | Urine | >8/4 R | >1 R | >8 R | >8 R | >4 R | >16 R | >1 R | >16/4 R | ≤1 S |
| Kleb_50 | Rectal swab | >8/4 R | >1 R | >8 R | >8 R | >4 R | >16 R | >1 R | >16/4 R | ≤1 S |
| Kleb_51 | Urine | >8/4 R | >1 R | >8 R | >8 R | ≤1 S | >8 R | >1 R | >16/4 R | ≤1 S |
| Kleb_52 | Blood culture | >8/4 R | >1 R | >16 R | >8 R | >4 R | >16 R | >1 R | >64/4 R | 0.5 S |
| Kleb_53 | Urine | >8/4 R | >1 R | >16 R | >8 R | 2 S | >16 R | >1 R | >64/4 R | 0.25 S |
| Kleb_54 | Rectal swab | 256/4 R | >1 R | >8 R | >8 R | >4 R | >16 R | >1 R | >16/4 R | ≤1 S |
| Kleb_55 | Rectal swab | 256/4 R | >1 R | 32 R | >8 R | ≤1 S | >8 R | >1 R | >64/4 R | 0.5 S |
| Kleb_56 | Urine | 256/4 R | >1 R | >8 R | >8 R | >4 R | >16 R | >1 R | >16/4 R | ≤1 S |
| Kleb_57 | Urine | >8/4 R | >1 R | >8 R | >8 R | >4 R | >16 R | >1 R | >16/4 R | ≤1 S |
| Kleb_58 | Rectal swab | 256/4 R | >1 R | >8 R | >8 R | >4 R | >16 R | >1 R | >16/4 R | 0.5 S |
| Kleb_59 | Rectal swab | >8/4 R | >1 R | >16 R | >8 R | >4 R | >16 R | >1 R | >64/4 R | 0.5 S |
| Kleb_60 | Urine | 256/4 R | >1 R | >16 R | >8 R | >4 R | >16 R | >1 R | >64/4 R | 0.5 S |
| Kleb_61 | Rectal swab | 256/4 R | >1 R | >8 R | >8 R | >4 R | >8 R | >1 R | >16/4 R | 0.5 S |
| Kleb_62 | Urine | 256/4 R | >1 R | >8 R | >8 R | >4 R | >16 R | >1 R | >16/4 R | 0.5 S |
| Kleb_63 | Urine | >8/4 R | >1 R | >8 R | >8 R | >4 R | >16 R | >1 R | >16/4 R | 0.5 S |
| Kleb_64 | Urine | >8/4 R | >1 R | >8 R | >8 R | >4 R | >16 R | >1 R | >16/4 R | 0.5 S |
| Kleb_65 | Rectal swab | >8/4 R | >1 R | >16 R | >8 R | >4 R | >16 R | >1 R | >64/4 R | 0.5 S |
| Kleb_66 | Rectal swab | >8/4 R | >1 R | >16 R | >8 R | >4 R | >16 R | >1 R | >64/4 R | 0.5 S |
| Kleb_67 | Rectal swab | >8/4 R | >1 R | >16 R | >8 R | >4 R | >16 R | >1 R | >64/4 R | 0.5 S |
| Kleb_68 | Blood culture | >8/4 R | >1 R | >8 R | >8 R | >8 R | >16 R | >1 R | >64/4 R | 0.5 S |
| Kleb_69 | Urine | >8/4 R | >1 R | >8 R | >8 R | 2 S | >8 R | >1 R | >64/4 R | >1 R |
| Kleb_70 | Rectal swab | 256/4 R | >1 R | >8 R | >8 R | >4 R | >16 R | >1 R | >16/4 R | ≤1 S |
| Kleb_71 | Rectal swab | >8/4 R | >1 R | >16 R | >8 R | 2 S | >16 R | >1 R | >16/4 R | ≤1 S |
| Kleb_72 | Blood culture | >8/4 R | >1 R | >16 R | >8 R | >4 R | >16 R | >1 R | >64/4 R | 0.5 S |
| Kleb_73 | Rectal swab | >8/4 R | >1 R | >16 R | >8 R | >32 R | >8 R | >1 R | >64/4 R | ≤1 S |
| Kleb_74 | Rectal swab | >8/4 R | >1 R | 64 R | >8 R | >8 R | >8 R | >1 R | >64/4 R | 0.5 S |
| Kleb_75 | Rectal swab | >8/4 R | >1 R | 32 R | >8 R | >8 R | >16 R | >1 R | >64/4 R | 0.5 S |
| Kleb_76 | Urine | >8/4 R | >1 R | 64 R | >8 R | >8 R | >16 R | >1 R | >64/4 R | 0.5 S |
| Kleb_77 | Rectal swab | >8/4 R | >1 R | >16 R | >8 R | 2 S | >32 R | >1 R | >16/4 R | 0.5 S |
| Kleb_78 | Rectal swab | >8/4 R | >1 R | >8 R | >8 R | >32 R | >8 R | >1 R | >64/4 R | ≤1 S |
| Kleb_79 | Rectal swab | >8/4 R | >1 R | >16 R | >8 R | >4 R | >16 R | >1 R | >64/4 R | ≤1 S |
| Kleb_80 | Rectal swab | >8/4 R | >1 R | 64 R | >8 R | >4 R | >16 R | >1 R | >64/4 R | 0.5 S |
| Kleb_81 | Rectal swab | 256/4 R | >1 R | >8 R | >8 R | >4 R | >8 R | >1 R | >16/4 R | 0.5 S |
| Kleb_82 | Rectal swab | 256/4 R | >1 R | >8 R | >8 R | >4 R | >16 R | >1 R | >16/4 R | 0.5 S |
| Kleb_83 | Rectal swab | 256/4 R | >1 R | >8 R | >8 R | >4 R | >16 R | >1 R | >16/4 R | 0.5 S |
| Kleb_84 | Rectal swab | >8/4 R | >1 R | >16 R | >8 R | ≥16 R | >8 R | >1 R | >64/4 R | 0.5 S |
| Kleb_85 | Blood culture | >8/4 R | >1 R | >16 R | ≥16 R | ≥16 R | >8 R | >1 R | >64/4 R | ≤1 S |
| Kleb_86 | Rectal swab | >8/4 R | >1 R | >16 R | >8 R | ≥16 R | >8 R | >1 R | >64/4 R | ≤1 S |
| Kleb_87 | Stool | 256/4 R | >1 R | >8 R | >8 R | 2 S | >16 R | >1 R | >16/4 R | >4 R |
| Kleb_88 | Rectal swab | >8/4 R | >1 R | >8 R | >8 R | >4 R | >16 R | >1 R | >16/4 R | ≤1 S |
| Kleb_89 | Rectal swab | >8/4 R | >1 R | >8 R | >8 R | >4 R | >16 R | >1 R | >16/4 R | ≤1 S |
| Kleb_90 | Blood culture | >8/4 R | >1 R | >8 R | >8 R | >4 R | >16 R | >1 R | >16/4 R | 0.5 S |
| Kleb_91 | Urine | >8/4 R | >1 R | >8 R | >8 R | >4 R | >16 R | >1 R | >16/4 R | 0.5 S |
| Kleb_92 | Urine | >8/4 R | >1 R | >8 R | >8 R | >4 R | >16 R | >1 R | >16/4 R | 0.5 S |
| Kleb_93 | Wound swab | >8/4 R | >1 R | >8 R | >8 R | >4 R | >16 R | >1 R | >16/4 R | 0.5 S |
| Kleb_94 | Urine | >8/4 R | >1 R | >8 R | >8 R | >4 R | >16 R | >1 R | >16/4 R | 0.5 S |
| Kleb_95 | Wound swab | >8/4 R | >1 R | >8 R | >8 R | >4 R | >16 R | >1 R | >16/4 R | 0.5 S |
| Kleb_96 | Rectal swab | >8/4 R | >1 R | >8 R | >8 R | >4 R | >16 R | >1 R | >16/4 R | 0.5 S |
| Kleb_97 | Rectal swab | >8/4 R | >1 R | >16 R | >8 R | >4 R | >16 R | >1 R | >16/4 R | 0.5 S |
| Kleb_98 | Rectal swab | >8/4 R | >1 R | >16 R | >8 R | ≥16 R | >8 R | >1 R | >64/4 R | ≤1 S |
| Kleb_99 | Rectal swab | >8/4 R | >1 R | >16 R | >8 R | ≥16 R | >32 R | >1 R | >16/4 R | 0.5 S |
| Kleb_100 | Urine | >8/4 R | >1 R | >8 R | >8 R | >4 R | >16 R | >1 R | >16/4 R | ≤1 S |
| Kleb_101 | Rectal swab | >8/4 R | >1 R | >16 R | ≥16 R | ≥16 R | >16 R | >1 R | >64/4 R | 0.5 S |
| Kleb_102 | Rectal swab | >8/4 R | >1 R | >16 R | ≥16 R | ≥16 R | >32 R | >1 R | >64/4 R | 0.5 S |
| Kleb_103 | Rectal swab | 256/4 R | >1 R | >8 R | >8 R | >4 R | >16 R | >1 R | >16/4 R | ≤1 S |
| Kleb_104 | Urine | 256/4 R | >1 R | >16 R | >8 R | ≥16 R | >8 R | >1 R | >64/4 R | 0.5 S |
| Kleb_105 | Urine | >8/4 R | >1 R | >16 R | ≥16 R | ≥16 R | >32 R | >1 R | >64/4 R | 0.5 S |
| Kleb_106 | Urine | >8/4 R | >1 R | >8 R | >8 R | >4 R | >16 R | >1 R | >16/4 R | ≤1 S |
| Kleb_107 | Urine | >8/4 R | >1 R | >8 R | >8 R | >4 R | >16 R | >1 R | >16/4 R | ≤1 S |
| Kleb_108 | Rectal swab | >8/4 R | >1 R | >8 R | >8 R | 2 S | >16 R | >1 R | >64/4 R | >4 R |
| Kleb_109 | Urine | >8/4 R | >1 R | >8 R | >8 R | >4 R | >16 R | >1 R | >16/4 R | ≤1 S |
| Kleb_110 | Rectal swab | >8/4 R | >1 R | >16 R | >8 R | >4 R | >16 R | >1 R | >16/4 R | 0.5 S |

**Table S1.** Antimicrobial profile of the isolates

Abbreviations: MIC, minimum inhibitory concentration; CZA, ceftazidime-avibactam; ERT, ertapenem; MEM, meropenem; IMI, imipenem; GENT, gentamicin; AMK, amikacin; CIP, ciprofloxacin; TZP, piperacillin-tazobactam; COL, colistin; BAL, bronchoalveolar lavage; S, susceptible; I, susceptible, increased exposure; R, resistant.
