# Supplementary material for: A rapid molecular assay for the detection of hypervirulent Klebsiella pneumoniae in the context of antimicrobial resistance surveillance
Source: Microbiol Spectr. 2026 Jul 27;14(9):e01068-26. doi: 10.1128/spectrum.01068-26 (PMC13532228; doi:10.1128/spectrum.01068-26)
Supplement: Supplementary file 2 [file spectrum.01068-26-s0002.docx]

# Table S2. Strain-by-strain comparison of WGS-derived virulence profiles and Real-Time PCR findings in 110 *Klebsiella pneumoniae* isolates, including sequence type and sequencing coverage.

| **Strain** | **WGS score** | **WGS *ybt*** | **WGS *iuc*** | **WGS *clb*** | **PCR *rmpA*** | **PCR *rmpA2*** | **PCR *rmpC*** | **PCR *ybtA*** | **PCR *ybtS*** | **PCR *iucA*** | **PCR *peg-344*** | **PCR *mrkD*** | **PCR *fimH*** | **Concordance** | **Notes** | **Sequence Type (ST)** | **Coverage** | **BioSample accession** |
| --- | --- | --- | --- | --- | --- | --- | --- | --- | --- | --- | --- | --- | --- | --- | --- | --- | --- | --- |
| Kleb_01 | 3 | − | + | − | + | + | − | − | − | + | − | + | + | Concordant |  | 1805 | 52x | SAMN45618068 |
| Kleb_02 | 4 | + | + | − | + | + | + | + | + | + | + | + | + | Concordant |  | 147 | 41x | SAMN45618069 |
| Kleb_03 | 4 | + | + | − | + | + | + | + | + | + | + | + | + | Concordant |  | 147 | 43x | SAMN45618073 |
| Kleb_04 | 4 | + | + | − | + | + | − | + | + | + | − | − | − | Concordant | PCR negative for *peg-344*, *fimH*, and *mrkD*. | 147 | 37x | SAMN45618077 |
| Kleb_05 | 3 | − | + | − | + | + | − | − | − | + | − | + | + | Concordant |  | 1805 | 41x | SAMN56274969 |
| Kleb_06 | 4 | + | + | − | + | + | + | + | + | + | + | + | + | Concordant |  | 147 | 45x | SAMN56274975 |
| Kleb_07 | 1 | + | − | − | − | − | − | + | + | − | − | + | + | Concordant |  | 147 | 92x | SAMN56402428 |
| Kleb_08 | 4 | + | + | − | + | + | + | + | + | + | + | + | + | Concordant |  | 147 | 55x | SAMN56402445 |
| Kleb_09 | 3 | − | + | − | + | + | − | − | − | + | − | + | + | Concordant |  | 1805 | 55x | SAMN56402461 |
| Kleb_10 | 0 | − | − | − | − | − | − | − | − | − | − | + | + | Concordant |  | 14 | 63x | SAMN56402462 |
| Kleb_11 | 4 | + | + | − | + | + | + | + | + | + | + | + | + | Concordant |  | 147 | 73x | SAMN56449418 |
| Kleb_12 | 4 | + | + | − | + | + | + | + | + | + | + | + | + | Concordant |  | 147 | 64x | SAMN56449428 |
| Kleb_13 | 4 | + | + | − | + | − | + | + | + | + | + | + | + | Concordant |  | 147 | 79x | SAMN56449429 |
| Kleb_14 | 1 | + | − | − | − | − | − | + | + | − | − | + | + | Concordant |  | 11 | 73x | SAMN56449430 |
| Kleb_15 | 4 | + | + | − | + | + | − | + | + | + | − | + | + | Concordant | PCR negative for *peg-344*. |  | 97x | SAMN56449432 |
| Kleb_16 | 4 | + | + | − | + | + | + | + | + | + | + | + | + | Concordant |  | 147 | 82x | SAMN56449433 |
| Kleb_17 | 4 | + | + | − | + | + | + | + | + | + | + | + | + | Concordant |  | 147 | 49x | SAMN56449434 |
| Kleb_18 | 4 | + | + | − | + | + | + | + | + | + | + | + | + | Concordant |  | 147 | 50x | SAMN56449435 |
| Kleb_19 | 3 | − | + | − | + | + | − | − | − | + | − | + | + | Concordant |  | 1805 | 72x | SAMN56449436 |
| Kleb_20 | 4 | + | + | − | + | + | + | + | + | + | + | + | + | Concordant |  | 147 | 67x | SAMN56449437 |
| Kleb_21 | 4 | + | + | − | + | + | + | + | + | + | + | + | + | Concordant |  | 147 | 67x | SAMN56449438 |
| Kleb_22 | 4 | + | + | − | + | + | + | + | + | + | + | + | + | Concordant |  | 147 | 62x | SAMN56449439 |
| Kleb_23 | 0 | − | − | − | − | − | − | − | − | − | − | + | + | Concordant |  | 200 | 70x | SAMN56449440 |
| Kleb_24 | 4 | + | + | − | + | + | + | + | + | + | + | + | + | Concordant |  | 147 | 65x | SAMN56449441 |
| Kleb_25 | 4 | + | + | − | + | + | − | + | + | + | − | + | + | Concordant | PCR negative for *peg-344*. | 147 | 87x | SAMN56449442 |
| Kleb_26 | 4 | + | + | − | + | + | + | + | + | + | + | + | + | Concordant |  | 147 | 58x | SAMN56449443 |
| Kleb_27 | 3 | − | + | − | + | + | − | + | + | + | − | + | + | Partially discordant | WGS score 3; PCR detected *ybtA*, *ybtS*, and *iucA*. | *475a | 54x | SAMN56449444 |
| Kleb_28 | 4 | + | + | − | + | + | + | + | + | + | + | + | + | Concordant |  | 147 | 58x | SAMN56449445 |
| Kleb_29 | 3 | − | + | − | + | + | − | + | − | + | − | + | + | Partially discordant | WGS score 3; PCR detected *ybtA* and *iucA*, but not *ybtS*. | *475a | 64x | SAMN56449446 |
| Kleb_30 | 4 | + | + | − | + | + | + | + | + | + | + | + | + | Concordant |  | 147 | 61x | SAMN56449447 |
| Kleb_31 | 4 | + | + | − | + | + | + | + | + | + | + | + | + | Concordant |  | 11 | 53x | SAMN56449448 |
| Kleb_32 | 4 | + | + | − | + | + | + | + | + | + | + | + | + | Concordant |  | 147 | 52x | SAMN56449449 |
| Kleb_33 | 1 | + | − | − | − | − | − | + | + | − | − | + | + | Concordant |  | 147 | 60x | SAMN56449453 |
| Kleb_34 | 4 | + | + | − | + | + | + | + | + | + | + | + | + | Concordant |  | 147 | 33x | SAMN56449454 |
| Kleb_35 | 4 | + | + | − | + | + | + | + | + | + | + | + | + | Concordant |  | 147 | 50x | SAMN56449455 |
| Kleb_36 | 3 | − | + | − | + | + | − | − | − | + | − | + | + | Concordant |  | 1805 | 69x | SAMN56449456 |
| Kleb_37 | 4 | + | + | − | + | + | + | + | + | + | + | + | + | Concordant |  | 147 | 59x | SAMN56449457 |
| Kleb_38 | 0 | − | − | − | − | − | − | − | − | − | − | + | + | Concordant |  | 11 | 31x | SAMN56449458 |
| Kleb_39 | 4 | + | + | − | + | + | + | + | + | + | + | + | + | Concordant |  | 147 | 32x | SAMN56449460 |
| Kleb_40 | 4 | + | + | − | + | + | + | + | + | + | + | + | + | Concordant |  | 147 | 58x | SAMN56449461 |
| Kleb_41 | 1 | + | − | − | − | − | − | + | + | − | − | + | + | Concordant |  | 147 | 38x | SAMN45618070 |
| Kleb_42 | 1 | + | − | − | − | − | − | + | + | − | − | + | + | Concordant |  | 11 | 40x | SAMN45618071 |
| Kleb_43 | 1 | + | − | − | − | − | − | + | + | − | − | + | + | Concordant |  | 11 | 48x | SAMN45618072 |
| Kleb_44 | 1 | + | − | − | − | − | − | + | + | − | − | + | + | Concordant |  | 147 | 75x | SAMN45618074 |
| Kleb_45 | 1 | + | − | − | − | − | − | + | + | − | − | − | − | Concordant |  | 147 | 58x | SAMN45618075 |
| Kleb_46 | 1 | + | − | − | − | − | − | + | + | − | − | + | + | Concordant |  | 147 | 24x | SAMN45618076 |
| Kleb_47 | 1 | + | − | − | − | − | − | + | + | − | − | + | + | Concordant |  | 147 | 31x | SAMN56274967 |
| Kleb_48 | 1 | + | − | − | − | − | − | + | + | − | − | + | + | Concordant |  | 11 | 77x | SAMN56274968 |
| Kleb_49 | 1 | + | − | − | − | − | − | + | + | − | − | + | + | Concordant |  | 11 | 48x | SAMN56274970 |
| Kleb_50 | 1 | + | − | − | − | − | − | − | − | − | − | + | + | Partially discordant |  | 147 | 47x | SAMN56274971 |
| Kleb_51 | 1 | + | − | − | − | − | − | + | + | − | − | + | + | Concordant |  | 11 | 63x | SAMN56274972 |
| Kleb_52 | 1 | + | − | − | − | − | − | − | − | − | − | + | + | Partially discordant |  | 147 | 58x | SAMN56274973 |
| Kleb_53 | 1 | + | − | − | − | − | − | + | + | − | − | + | + | Concordant |  | 147 | 58x | SAMN56274974 |
| Kleb_54 | 1 | + | − | − | − | − | − | + | + | − | − | + | + | Concordant |  | 147 | 50x | SAMN56274976 |
| Kleb_55 | 1 | + | − | − | − | − | − | + | + | − | − | + | + | Concordant |  | 147 | 66x | SAMN56402426 |
| Kleb_56 | 1 | + | − | − | − | − | − | + | + | − | − | + | + | Concordant |  | 147 | 65x | SAMN56402427 |
| Kleb_57 | 1 | + | − | − | − | − | − | + | + | − | − | + | + | Concordant |  | 147 | 58x | SAMN56402429 |
| Kleb_58 | 1 | + | − | − | − | − | − | + | + | − | − | + | + | Concordant |  | 11 | 27x | SAMN56402430 |
| Kleb_59 | 1 | + | − | − | − | − | − | + | + | − | − | + | + | Concordant |  | 147 | 41x | SAMN56402431 |
| Kleb_60 | 1 | + | − | − | − | − | − | + | + | − | − | + | + | Concordant |  | 147 | 30x | SAMN56402432 |
| Kleb_61 | 1 | + | − | − | − | − | − | + | + | − | − | + | + | Concordant |  | 512 | 26x | SAMN56402433 |
| Kleb_62 | 1 | + | − | − | − | − | − | + | + | − | − | + | + | Concordant |  | 147 | 44x | SAMN56402434 |
| Kleb_63 | 1 | + | − | − | − | − | − | + | + | − | − | + | + | Concordant |  | 147 | 51x | SAMN56402435 |
| Kleb_64 | 1 | + | − | − | − | − | − | + | + | − | − | + | + | Concordant |  | 147 | 52x | SAMN56402436 |
| Kleb_65 | 1 | + | − | − | − | − | − | + | + | − | − | + | + | Concordant |  | 147 | 102x | SAMN56402437 |
| Kleb_66 | 1 | + | − | − | − | − | − | + | + | − | − | + | + | Concordant |  | 147 | 91x | SAMN56402438 |
| Kleb_67 | 1 | + | − | − | − | − | − | + | + | − | − | + | + | Concordant |  | 147 | 124x | SAMN56402439 |
| Kleb_68 | 1 | + | − | − | − | − | − | + | + | − | − | + | + | Concordant |  | 512 | 71x | SAMN56402440 |
| Kleb_69 | 1 | + | − | − | − | − | − | − | − | − | − | − | − | Partially discordant |  | 147 | 79x | SAMN56402441 |
| Kleb_70 | 1 | + | − | − | − | − | − | + | − | − | − | + | + | Concordant |  | 147 | 112x | SAMN56402442 |
| Kleb_71 | 1 | + | − | − | − | − | − | + | + | − | − | + | + | Concordant |  | 147 | 88x | SAMN56402443 |
| Kleb_72 | 1 | + | − | − | − | − | − | + | + | − | − | − | − | Concordant |  | 147 | 168x | SAMN56402444 |
| Kleb_73 | 1 | + | − | − | − | − | − | + | + | − | − | − | − | Concordant |  | 147 | 65x | SAMN56402446 |
| Kleb_74 | 1 | + | − | − | − | − | − | − | − | − | − | − | − | Partially discordant |  | 11 | 46x | SAMN56402447 |
| Kleb_75 | 1 | + | − | − | − | − | − | + | + | − | − | − | − | Concordant |  | 11 | 62x | SAMN56402448 |
| Kleb_76 | 1 | + | − | − | − | − | − | + | + | − | − | + | + | Concordant |  | 512 | 68x | SAMN56402449 |
| Kleb_77 | 1 | + | − | − | − | − | − | + | + | − | − | − | − | Concordant |  | 147 | 68x | SAMN56402450 |
| Kleb_78 | 1 | + | − | − | − | − | − | + | + | − | − | − | − | Concordant |  | 11 | 50x | SAMN56402451 |
| Kleb_79 | 1 | + | − | − | − | − | − | + | + | − | − | − | − | Concordant |  | 147 | 59x | SAMN56402452 |
| Kleb_80 | 1 | + | − | − | − | − | − | + | + | − | − | − | − | Concordant |  | 147 | 49x | SAMN56402453 |
| Kleb_81 | 1 | + | − | − | − | − | − | + | + | − | − | − | − | Concordant |  | 512 | 57x | SAMN56402454 |
| Kleb_82 | 1 | + | − | − | − | − | − | + | + | − | − | − | − | Concordant |  | 147 | 44x | SAMN56402455 |
| Kleb_83 | 1 | + | − | − | − | − | − | − | − | − | − | − | − | Partially discordant |  | 147 | 71x | SAMN56402456 |
| Kleb_84 | 1 | + | − | − | − | − | − | − | − | − | − | − | − | Partially discordant |  | 147 | 49x | SAMN56402457 |
| Kleb_85 | 1 | + | − | − | − | − | − | − | − | − | − | − | − | Partially discordant |  | 147 | 44x | SAMN56402458 |
| Kleb_86 | 1 | + | − | − | − | − | − | − | − | − | − | − | − | Partially discordant |  | 147 | 53x | SAMN56402459 |
| Kleb_87 | 1 | + | − | − | − | − | − | − | − | − | − | − | − | Partially discordant |  | 147 | 76x | SAMN56402460 |
| Kleb_88 | 1 | + | − | − | − | − | − | + | + | − | − | − | − | Concordant |  | 11 | 90x | SAMN56402463 |
| Kleb_89 | 1 | + | − | − | − | − | − | + | + | − | − | − | − | Concordant |  | 11 | 86x | SAMN56402464 |
| Kleb_90 | 1 | + | − | − | − | − | − | − | − | − | − | − | − | Partially discordant |  | 147 | 65x | SAMN56402465 |
| Kleb_91 | 1 | + | − | − | − | − | − | − | − | − | − | − | − | Partially discordant |  | 147 | 66x | SAMN56402466 |
| Kleb_92 | 1 | + | − | − | − | − | − | + | + | − | − | − | − | Concordant |  | 147 | 64x | SAMN56402467 |
| Kleb_93 | 1 | + | − | − | − | − | − | − | − | − | − | − | − | Partially discordant |  | 147 | 60x | SAMN56402468 |
| Kleb_94 | 1 | + | − | − | − | − | − | − | − | − | − | − | − | Partially discordant |  | 147 | 151x | SAMN56402469 |
| Kleb_95 | 1 | + | − | − | − | − | − | − | − | − | − | + | + | Partially discordant |  | 147 | 67x | SAMN56402470 |
| Kleb_96 | 1 | + | − | − | − | − | − | − | − | − | − | + | + | Partially discordant |  | 11 | 41x | SAMN56449419 |
| Kleb_97 | 1 | + | − | − | − | − | − | − | − | − | − | + | + | Partially discordant |  | 11 | 90x | SAMN56449420 |
| Kleb_98 | 1 | + | − | − | − | − | − | − | − | − | − | − | − | Partially discordant |  | 147 | 94x | SAMN56449421 |
| Kleb_99 | 1 | + | − | − | − | − | − | − | − | − | − | − | − | Partially discordant |  | 11 | 45x | SAMN56449422 |
| Kleb_100 | 1 | + | − | − | − | − | − | + | + | − | − | + | + | Concordant |  | 11 | 107x | SAMN56449423 |
| Kleb_101 | 1 | + | − | − | − | − | − | + | + | − | − | − | − | Concordant |  | 11 | 62x | SAMN56449424 |
| Kleb_102 | 1 | + | − | − | − | − | − | + | + | − | − | + | + | Concordant |  | 147 | 47x | SAMN56449425 |
| Kleb_103 | 1 | + | − | − | − | − | − | + | + | − | − | + | + | Concordant |  | 147 | 106x | SAMN56449426 |
| Kleb_104 | 1 | + | − | − | − | − | − | + | + | − | − | + | + | Concordant |  | 11 | 19x | SAMN56449427 |
| Kleb_105 | 0 | − | − | − | − | − | − | − | − | − | − | + | + | Concordant |  | 11 | 111x | SAMN56449431 |
| Kleb_106 | 0 | − | − | − | − | − | − | − | − | − | − | + | + | Concordant |  | 20 | 75x | SAMN56449450 |
| Kleb_107 | 0 | − | − | − | − | − | − | − | − | − | − | + | + | Concordant |  | 20 | 68x | SAMN56449451 |
| Kleb_108 | 0 | − | − | − | − | − | − | − | − | − | − | − | − | Concordant |  | 147 | 53x | SAMN56449452 |
| Kleb_109 | 0 | − | − | − | − | − | − | + | − | − | − | + | + | Partially discordant |  | 39 | 68x | SAMN56449459 |
| Kleb_110 | 0 | − | − | − | − | − | − | − | − | − | − | + | + | Concordant |  | 147 | 84x | SAMN56449462 |

**Abbreviations.** WGS, whole-genome sequencing; PCR, polymerase chain reaction; *ybt*, yersiniabactin locus; *iuc*, aerobactin locus; *clb*, colibactin locus.

**Legend.** Presence (+) or absence (−) of virulence determinants detected by WGS and Real-Time PCR for each isolate included in the study. WGS virulence score was assigned using Kleborate. According to the scoring system, scores were interpreted as follows: 0 = no virulence genes; 1 = *ybt*; 2 = *ybt* and *clb*; 3 = *iuc*; 4 = *ybt* and *iuc*; 5 = *ybt*, *clb*, and *iuc*.

Supplementary Table S2 also reports sequence type (ST) and sequencing coverage for each isolate to facilitate interpretation of concordant and discordant findings between the two methods.

* ST assignments marked with an asterisk indicate provisional sequence-type calls and should be interpreted with caution.

Genome coverage was estimated as the ratio between the total number of sequenced bases and the reference *Klebsiella* *pneumoniae* genome size (5.5 Mb), and is reported as ×-fold coverage.
