# Supplementary material for: A rapid molecular assay for the detection of hypervirulent Klebsiella pneumoniae in the context of antimicrobial resistance surveillance
Source: Microbiol Spectr. 2026 Jul 27;14(9):e01068-26. doi: 10.1128/spectrum.01068-26 (PMC13532228; doi:10.1128/spectrum.01068-26)
Supplement: Supplementary file 3 [file spectrum.01068-26-s0003.docx]

**Table S3**. *Resistance genes identified through WGS analysis and corresponding virulence scores^1^*

| **Strain** | **Resistance genes from WGS** | **Virulence score from WGS** | **Virulence genes by Real-Time PCR** |
| --- | --- | --- | --- |
| Kleb_01 | *bla*_NDM-1_, *bla*_CTX-M-15_, *bla*_OXA-1_, *bla*_TEM-1D_, *bla*_SHV-27_ | 3 | *rmpA2, iucA, mrkD, fimH* |
| Kleb_02 | *bla*_NDM-1_, *bla*_CTX-M-15_, *bla*_OXA-1_, *bla*_OXA-9_, *bla*_TEM-1D_, *bla*_SHV-11_ | 4 | *rmpA2, rmpC, rmpA, ybtA, iucA, ybtS, peg-344, mrkD, fimH* |
| Kleb_03 | *bla*_NDM-1_, *bla*_CTX-M-15_, *bla*_OXA-1_, *bla*_OXA-9_, *bla*_TEM-1D_, *bla*_SHV-11_ | 4 | *rmpA2, rmpC, rmpA, ybtA, iucA, ybtS, peg-344, mrkD, fimH* |
| Kleb_04 | *bla*_NDM-5_, *bla*_CTX-M-15_, *bla*_SHV-11_, *bla*_CMY-2_ | 4 | *rmpA2, rmpA, ybtA, iucA, ybtS* |
| Kleb_05 | *bla*_NDM-1_ | 3 | *rmpA2, iucA, fimH, mrkD* |
| Kleb_06 | *bla*_NDM-1_, *bla*_CTX-M-15_, *bla*_OXA-1_, *bla*_OXA-9_, *bla*_TEM-1D_, *bla*_SHV-11_ | 4 | *rmpA2, rmpC, rmpA, ybtA, iucA, ybtS, peg-344, mrkD, fimH* |
| Kleb_07 | *bla*_NDM-1_, *bla*_OXA-48_, *bla*_CTX-M-15_, *bla*_SHV-11_ | 1 | *ybtA, ybtS, mrkD, fimH* |
| Kleb_08 | *bla*_NDM-1_, *bla*_CTX-M-15_, *bla*_OXA-1_, *bla*_OXA-9,_ *bla*_TEM-1D,_ *bla*_SHV-11_ | 4 | *rmpA2, rmpC, rmpA, ybtA, iucA, ybtS, peg-344, mrkD, fimH* |
| Kleb_09 | *bla*_NDM-1_ | 3 | *rmpA2, iucA, mrkD, fimH* |
| Kleb_10 | *bla*_NDM-5,_ *bla*_OXA-10,_ *bla*_SHV-28_ | 0 | *mrkD, fimH* |
| Kleb_11 | *bla*_NDM-1_, *bla*_CTX-M-15_, *bla*_OXA-9_, *bla*_OXA-1_, *bla*_TEM-1D,_ *bla*_SHV-11_ | 4 | *rmpA2, rmpC, rmpA, ybtA, iucA, ybtS, peg-344, mrkD, fimH* |
| Kleb_12 | *bla*_NDM-1_, *bla*_CTX-M-15_, *bla*_OXA-1_, *bla*_OXA-9_, *bla*_TEM-1D,_ *bla*_SHV-11_ | 4 | *rmpA2, rmpC, rmpA, ybtA, iucA, ybtS, peg-344, mrkD, fimH* |
| Kleb_13 | *bla*_NDM-5_, *bla*_OXA-48_, *bla*_CTX-M-14_, *bla*_CTX-M-15_, *bla*_OXA-1_, *bla*_TEM-1D,_ *bla*_SHV-11_ | 4 | *rmpC, rmpA, ybtA, iucA, ybtS, peg-344, mrkD, fimH* |
| Kleb_14 | *bla*_NDM-1_, *bla*_CTX-M-15_, *bla*_OXA-1_, *bla*_SHV-11_ | 1 | *ybtA, ybtS, mrkD, fimH* |
| Kleb_15 | *la_KPC-3_, bla_SHV-1_* | 4 | *rmpA2, ybtA, iucA, ybtS, mrkD, fimH* |
| Kleb_16 | *bla*_NDM-1_, *bla*_CTX-M-15,_ *bla*_OXA-1_, *bla*_OXA-9_, *bla*_TEM-1D,_ *bla*_SHV-11_ | 4 | *rmpA2, rmpC, rmpA, ybtA, iucA, ybtS, peg-344, mrkD, fimH* |
| Kleb_17 | *bla*_NDM-1_, *bla*_CTX-M-15,_ *bla*_OXA-1_, *bla*_OXA-9_, *bla*_TEM-1D,_ *bla*_SHV-11_ | 4 | *rmpA2, rmpC, rmpA, ybtA, iucA, ybtS, peg-344, mrkD, fimH* |
| Kleb_18 | *bla*_NDM-1_, *bla*_CTX-M-15,_ *bla*_OXA-1_, *bla*_OXA-9_, *bla*_TEM-1D,_ *bla*_SHV-11_ | 4 | *rmpA2, rmpC, rmpA, ybtA, iucA, ybtS, peg-344, mrkD, fimH* |
| Kleb_19 | *bla*_NDM-1_, *bla*_SHV-27_ | 3 | *rmpA2, iucA, mrkD, fimH* |
| Kleb_20 | *bla*_NDM-1_, *bla*_CTX-M-15,_ *bla*_OXA-1_, *bla*_OXA-9_, *bla*_TEM-1D,_ *bla*_SHV-11_ | 4 | *rmpA2, rmpC, rmpA, ybtA, iucA, ybtS, peg-344, mrkD, fimH* |
| Kleb_21 | *bla*_NDM-1_, *bla*_CTX-M-15,_ *bla*_OXA-1_, *bla*_OXA-9_, *bla*_TEM-1D,_ *bla*_SHV-11_ | 4 | *rmpA2, rmpC, rmpA, ybtA, iucA, ybtS, peg-344, mrkD, fimH* |
| Kleb_22 | *bla*_NDM-1_, *bla*_CTX-M-15,_ *bla*_OXA-1_, *bla*_OXA-9_, *bla*_TEM-1D,_ *bla*_SHV-11_ | 4 | *rmpA2, rmpC, rmpA, ybtA, iucA, ybtS, peg-344, mrkD, fimH* |
| Kleb_23 | *bla*_NDM-1_, *bla*_CTX-M-15,_ *bla*_OXA-10_, *bla*_SHV-1_ | 0 | *mrkD, fimH* |
| Kleb_24 | *bla*_NDM-1_, *bla*_CTX-M-15,_ *bla*_OXA-1_, *bla*_OXA-9_, *bla*_TEM-1D,_ *bla*_SHV-11_ | 4 | *rmpA2, rmpC, rmpA, ybtA, iucA, ybtS, peg-344, mrkD, fimH* |
| Kleb_25 | *bla*_NDM-5_, *bla*_CTX-M-15,_ *bla*_CMY-2,_ *bla*_TEM-1D,_ *bla*_SHV-11_ | 4 | *rmpA2, rmpA, ybtA, iucA, ybtS, mrkD, fimH* |
| Kleb_26 | *bla*_NDM-1_, *bla*_CTX-M-15,_ *bla*_OXA-1,_ *bla*_OXA-9,_ *bla*_TEM-1D,_ *bla*_SHV-11_ | 4 | *rmpA2, rmpC, rmpA, ybtA, iucA, ybtS, peg-344, mrkD, fimH* |
| Kleb_27 | *bla*_NDM-5_, *bla*_OXA-48_, *bla*_CTX-M-15,_ *bla*_TEM-1,_ *bla*_SHV-1_ | 3 | *rmpA2, ybtA, iucA, ybtS, mrkD, fimH* |
| Kleb_28 | *bla*_NDM-1_, *bla*_CTX-M-15,_ *bla*_OXA-1_, *bla*_OXA-9_, *bla*_TEM-1D,_ *bla*_SHV-11_ | 4 | *rmpA2, rmpC, rmpA, ybtA, iucA, ybtS, peg-344, mrkD, fimH* |
| Kleb_29 | *bla*_NDM-5_, *bla*_OXA-48_, *bla*_CTX-M-15,_ *bla*_TEM-1,_ *bla*_SHV-1_ | 3 | *rmpA2, ybtA, iucA, mrkD, fimH* |
| Kleb_30 | *bla*_NDM-1_, *bla*_CTX-M-15,_ *bla*_OXA-1_, *bla*_OXA-9_, *bla*_TEM-1D,_ *bla*_SHV-11_ | 4 | *rmpA2, rmpC, rmpA, ybtA, iucA, ybtS, peg-344, mrkD, fimH* |
| Kleb_31 | *bla*_NDM-1_, *bla*_KPC-2,_ *bla*_CTX-M-15,_ *bla*_SHV-11_ | 4 | *rmpA2, rmpC, rmpA, ybtA, iucA, ybtS, peg-344, mrkD, fimH* |
| Kleb_32 | *bla*_NDM-1_, *bla*_CTX-M-15,_ *bla*_OXA-1_, *bla*_OXA-9_, *bla*_TEM-1D,_ *bla*_SHV-11_ | 4 | *rmpA2, rmpC, rmpA, ybtA, iucA, ybtS, peg-344, mrkD, fimH* |
| Kleb_33 | *bla*_NDM-1_, *bla*_OXA-48_, *bla*_CTX-M-15,_ *bla*_SHV-11_ | 1 | *ybtA, ybtS, mrkD, fimH* |
| Kleb_34 | *bla*_NDM-5_, *bla*_OXA-48_, *bla*_OXA-1_, *bla*_TEM-1D,_ *bla*_CTX-M-14,_ *bla*_CTX-M-15,_ *bla*_SHV-11_ | 4 | *rmpA2, rmpC, rmpA, ybtA, iucA, ybtS, peg-344, mrkD, fimH* |
| Kleb_35 | *bla*_NDM-1_, *bla*_OXA-1_, *bla*_OXA-9_, *bla*_CTX-M-15,_ *bla*_TEM-1D,_ *bla*_SHV-11_ | 4 | *rmpA2, rmpC, rmpA, ybtA, iucA, ybtS, peg-344, mrkD, fimH* |
| Kleb_36 | *bla*_NDM-1_ | 3 | *rmpA2, iucA, mrkD, fimH* |
| Kleb_37 | *bla*_NDM-1_, *bla*_CTX-M-15,_ *bla*_OXA-1_, *bla*_OXA-9_, *bla*_TEM-1D,_ *bla*_SHV-11_ | 4 | *rmpA2, rmpC, rmpA, ybtA, iucA, ybtS, peg-344, mrkD, fimH* |
| Kleb_38 | *bla*_NDM-1_, *bla*_CTX-M-15,_ *bla*_OXA-1_, *bla*_TEM-1D,_ *bla*_SHV-11_ | 0 | *mrkD, fimH* |
| Kleb_39 | *bla*_NDM-1_, *bla*_CTX-M-15,_ *bla*_OXA-1_, *bla*_OXA-9_, *bla*_TEM-1D_, *bla*_SHV-11_ | 4 | *rmpA2, rmpC, rmpA, ybtA, iucA, ybtS, peg-344, mrkD, fimH* |
| Kleb_40 | *bla*_NDM-1_, *bla*_CTX-M-15,_ *bla*_OXA-1_, *bla*_OXA-9_, *bla*_TEM-1D_, *bla*_SHV-11_ | 4 | *rmpA2, rmpC, rmpA, ybtA, iucA, ybtS, peg-344, mrkD, fimH* |
| Kleb_41 | *bla*_NDM-1_, *bla*_OXA-48_, *bla*_CTX-M-15_, *bla*_SHV-11_ | 1 | *ybtA, ybtS, mrkD, fimH* |
| Kleb_42 | *bla*_NDM-1_, *bla*_KPC-3_, *bla*_CTX-M-15_, *bla*_OXA-1_, *bla*_TEM-1D_, *bla*_SHV-11_ | 1 | *ybtA, ybtS, mrkD, fimH* |
| Kleb_43 | *bla*_NDM-1_, *bla*_TEM-1D_, *bla*_SHV-11_ | 1 | *ybtA, ybtS, mrkD, fimH* |
| Kleb_44 | *bla*_NDM-1_, *bla*_OXA-48_, *bla*_CTX-M-15_, *bla*_SHV-11_ | 1 | *ybtA, ybtS, mrkD, fimH* |
| Kleb_45 | *bla*_NDM-1_, *bla*_OXA-48_, *bla*_CTX-M-15_, *bla*_SHV-11_ | 1 | *ybtA, ybtS* |
| Kleb_46 | *bla*_NDM-1_, *bla*_VIM-1_, *bla*_CTX-M-15_, *bla*_OXA-9_, *bla*_TEM-1D_ | 1 | *ybtA, ybtS, mrkD, fimH* |
| Kleb_47 | *bla*_NDM-1_, *bla*_VIM-1_, *bla*_CTX-M-15_, *bla*_OXA-9_, *bla*_TEM-1D_, *bla*_SHV-11_ | 1 | *ybtA, ybtS, mrkD, fimH* |
| Kleb_48 | *bla*_NDM-1_, *bla*_CTX-M-15_, *bla*_OXA-1_, *bla*_SHV-11_ | 1 | *ybtA, ybtS, mrkD, fimH* |
| Kleb_49 | *bla*_NDM-1_, *bla*_CTX-M-15_, *bla*_OXA-1_, *bla*_SHV-11_ | 1 | *ybtA, ybtS, mrkD, fimH* |
| Kleb_50 | *bla*_NDM-1_, *bla*_OXA-48_, *bla*_CTX-M-15_, *bla*_SHV-11_ | 1 | *mrkD, fimH* |
| Kleb_51 | *bla*_NDM-1_, *bla*_CTX-M-15_, *bla*_OXA-1_ | 1 | *ybtA, ybtS, mrkD, fimH* |
| Kleb_52 | *bla*_NDM-1_, *bla*_OXA-48_, *bla*_CTX-M-15_, *bla*_SHV-11_ | 1 | *mrkD, fimH* |
| Kleb_53 | *bla*_NDM-1_, *bla*_VIM-1_, *bla*_CTX-M-15_, *bla*_OXA-9_, *bla*_TEM-1D_ | 1 | *ybtA, ybtS, mrkD, fimH* |
| Kleb_54 | *bla*_NDM-1_, *bla*_OXA-48_, *bla*_CTX-M-15_, *bla*_SHV-11_ | 1 | *ybtA, ybtS, mrkD, fimH* |
| Kleb_55 | *bla*_NDM-1_, *bla*_VIM-1_, *bla*_CTX-M-15_, *bla*_OXA-9_, *bla*_TEM-1D_, *bla*_SHV-11_ | 1 | *ybtA, ybtS, mrkD, fimH* |
| Kleb_56 | *bla*_NDM-1_, *bla*_OXA-48_, *bla*_CTX-M-15_, *bla*_SHV-11_ | 1 | *ybtA, ybtS, mrkD, fimH* |
| Kleb_57 | *bla*_NDM-1_, *bla*_OXA-48_, *bla*_CTX-M-15_, *bla*_SHV-11_ | 1 | *ybtA, ybtS, mrkD, fimH* |
| Kleb_58 | *bla*_NDM-1_, *bla*_CTX-M-15_, *bla*_OXA-1_, *bla*_SHV-11_ | 1 | *ybtA, ybtS, mrkD, fimH* |
| Kleb_59 | *bla*_NDM-1_, *bla*_VIM-1_, *bla*_CTX-M-15_, *bla*_OXA-9_, *bla*_TEM-1D_ | 1 | *ybtA, ybtS, mrkD, fimH* |
| Kleb_60 | *bla*_NDM-1_, *bla*_OXA-48_, *bla*_CTX-M-15_, *bla*_SHV-11_ | 1 | *ybtA, ybtS, mrkD, fimH* |
| Kleb_61 | *bla*_NDM-5_, *bla*_KPC-3_, *bla*_SHV-11_ | 1 | *ybtA, ybtS, mrkD, fimH* |
| Kleb_62 | *bla*_NDM-1_, *bla*_OXA-48_, *bla*_CTX-M-15_, *bla*_SHV-11_ | 1 | *ybtA, ybtS, mrkD, fimH* |
| Kleb_63 | *bla*_NDM-1_, *bla*_OXA-48_, *bla*_CTX-M-15_, *bla*_SHV-11_ | 1 | *ybtA, ybtS, mrkD, fimH* |
| Kleb_64 | *bla*_NDM-1_, *bla*_OXA-48_, *bla*_CTX-M-15_, *bla*_SHV-11_ | 1 | *ybtA, ybtS, mrkD, fimH* |
| Kleb_65 | *bla*_NDM-1_, *bla*_OXA-48_, *bla*_CTX-M-15_, *bla*_SHV-11_ | 1 | *ybtA, ybtS, mrkD, fimH* |
| Kleb_66 | *bla*_NDM-1_, *bla*_OXA-48_, *bla*_CTX-M-15_, *bla*_SHV-11_ | 1 | *ybtA, ybtS, mrkD, fimH* |
| Kleb_67 | *bla*_NDM-1_, *bla*_OXA-48_, *bla*_CTX-M-15_, *bla*_SHV-11_ | 1 | *ybtA, ybtS, mrkD, fimH* |
| Kleb_68 | *bla*_NDM-1_, *bla*_CTX-M-15_, *bla*_OXA-1_, *bla*_TEM-1D_, *bla*_SHV-11_ | 1 | *ybtA, ybtS, mrkD, fimH* |
| Kleb_69 | *bla*_NDM-1_, *bla*_VIM-1_, *bla*_CTX-M-15_, *bla*_OXA-9_, *bla*_TEM-1D,_ *bla*_SHV-11_ | 1 | *-* |
| Kleb_70 | *bla*_NDM-1_, *bla*_OXA-48_, *bla*_CTX-M-15_, *bla*_SHV-11_ | 1 | *ybtA, mrkD, fimH* |
| Kleb_71 | *bla*_NDM-1_, *bla*_VIM-1_, *bla*_CTX-M-15_, *bla*_OXA-9_, *bla*_TEM-1D_ | 1 | *ybtA, ybtS, mrkD, fimH* |
| Kleb_72 | *bla*_NDM-1_, *bla*_OXA-48_, *bla*_CTX-M-15_, *bla*_SHV-11_ | 1 | *ybtA, ybtS* |
| Kleb_73 | *bla*_NDM-1_, *bla*_VIM-1_, *bla*_CTX-M-15_, *bla*_OXA-9_, *bla*_TEM-1D_ | 1 | *ybtA, ybtS* |
| Kleb_74 | *bla*_NDM-1_, *bla*_CTX-M-15_, *bla*_OXA-1,_ *bla*_SHV-11_ | 1 | *-* |
| Kleb_75 | *bla*_NDM-1_, *bla*_CTX-M-15_, *bla*_OXA-1_, *bla*_SHV-11_ | 1 | *ybtA, ybtS* |
| Kleb_76 | *bla*_NDM-1_, *bla*_CTX-M-15_, *bla*_OXA-1_, *bla*_TEM-1D,_ *bla*_SHV-11_ | 1 | *ybtA, ybtS, mrkD, fimH* |
| Kleb_77 | *bla*_NDM-1_, *bla*_OXA-48_, *bla*_CTX-M-15_, *bla*_SHV-11_ | 1 | *ybtA, ybtS* |
| Kleb_78 | *bla*_NDM-1_, *bla*_CTX-M-15_, *bla*_OXA-1_, *bla*_SHV-11_ | 1 | *ybtA, ybtS* |
| Kleb_79 | *bla*_NDM-1_, *bla*_VIM-1_, *bla*_CTX-M-15_, *bla*_OXA-9_, *bla*_TEM-1D,_ *bla*_SHV-11_ | 1 | *ybtA, ybtS* |
| Kleb_80 | *bla*_NDM-1_, *bla*_OXA-48_, *bla*_CTX-M-15_, *bla*_SHV-11_ | 1 | *ybtA, ybtS* |
| Kleb_81 | *bla*_NDM-5_, *bla*_KPC-3_, *bla*_SHV-11,_ *bla*_TEM-122_ | 1 | *ybtA, ybtS* |
| Kleb_82 | *bla*_NDM-1_, *bla*_OXA-48_, *bla*_CTX-M-15_, *bla*_SHV-11_ | 1 | *ybtA, ybtS* |
| Kleb_83 | *bla*_NDM-1_, *bla*_OXA-48_, *bla*_CTX-M-15_, *bla*_SHV-11_ | 1 | *-* |
| Kleb_84 | *bla*_NDM-1_, *bla*_OXA-48_, *bla*_CTX-M-15_, *bla*_SHV-11_ | 1 | *-* |
| Kleb_85 | *bla*_NDM-1_, *bla*_OXA-48_, *bla*_CTX-M-15_, *bla*_SHV-11_ | 1 | *-* |
| Kleb_86 | *bla*_NDM-1_, *bla*_VIM-1_, *bla*_CTX-M-15_, *bla*_OXA-9_, *bla*_TEM-1D_ | 1 | *-* |
| Kleb_87 | *bla*_NDM-1_, *bla*_CTX-M-15_, *bla*_OXA-9_, *bla*_TEM-1D,_ *bla*_SHV-11_ | 1 | *-* |
| Kleb_88 | *bla*_NDM-1_, *bla*_CTX-M-15_, *bla*_OXA-1_, *bla*_SHV-11_ | 1 | *ybtA, ybtS* |
| Kleb_89 | *bla*_NDM-1_, *bla*_CTX-M-15_, *bla*_OXA-1_, *bla*_SHV-11_ | 1 | *ybtA, ybtS* |
| Kleb_90 | *bla*_NDM-1_, *bla*_VIM-1_, *bla*_CTX-M-15_, *bla*_OXA-9_, *bla*_TEM-1D,_ *bla*_SHV-11_ | 1 | *-* |
| Kleb_91 | *bla*_NDM-1_, *bla*_VIM-1_, *bla*_CTX-M-15_, *bla*_SHV-12,_ *bla*_OXA-9_, *bla*_TEM-1D_ | 1 | *-* |
| Kleb_92 | *bla*_NDM-1_, *bla*_VIM-1_, *bla*_CTX-M-15_, *bla*_OXA-9_, *bla*_TEM-1D_ | 1 | *ybtA, ybtS* |
| Kleb_93 | *bla*_NDM-5,_ *bla*_CTX-M-15_, *bla*_OXA-9_, *bla*_OXA-1_, *bla*_TEM-1D,_ *bla*_SHV-11_ | 1 | *-* |
| Kleb_94 | *bla*_NDM-1_, *bla*_OXA-48_, *bla*_CTX-M-15_, *bla*_SHV-11_ | 1 | *-* |
| Kleb_95 | *bla*_NDM-1_, *bla*_OXA-48_, *bla*_CTX-M-15_, *bla*_SHV-11_ | 1 | *mrkD, fimH* |
| Kleb_96 | *bla*_NDM-1_, *bla*_CTX-M-15_, *bla*_OXA-1_, *bla*_SHV-11_ | 1 | *mrkD, fimH* |
| Kleb_97 | *bla*_NDM-1_, *bla*_CTX-M-15_, *bla*_OXA-1_, *bla*_SHV-11_ | 1 | *mrkD, fimH* |
| Kleb_98 | *bla*_NDM-1_, *bla*_OXA-48_, *bla*_CTX-M-15_, *bla*_SHV-11_ | 1 | *-* |
| Kleb_99 | *bla*_NDM-1_, *bla*_CTX-M-15_, *bla*_OXA-1_, *bla*_SHV-11_ | 1 | *-* |
| Kleb_100 | *bla*_NDM-1_, *bla*_CTX-M-15_, *bla*_OXA-1_, *bla*_SHV-11_ | 1 | *ybtA, ybtS, mrkD, fimH* |
| Kleb_101 | *bla*_NDM-1_, *bla*_CTX-M-15_, *bla*_OXA-1_, *bla*_SHV-11_ | 1 | *ybtA, ybtS* |
| Kleb_102 | *bla*_NDM-1_, *bla*_VIM-1_, *bla*_CTX-M-189_, *bla*_OXA-9_, *bla*_TEM-1D_ | 1 | *ybtA, ybtS, mrkD, fimH* |
| Kleb_103 | *bla*_NDM-1_, *bla*_OXA-48_, *bla*_CTX-M-15_, *bla*_SHV-11_ | 1 | *ybtA, ybtS, mrkD, fimH* |
| Kleb_104 | *bla*_NDM-1_, *bla*_CTX-M-15_, *bla*_OXA-1_, *bla*_SHV-11_ | 1 | *ybtA, ybtS, mrkD, fimH* |
| Kleb_105 | *bla*_NDM-1_, *bla*_CTX-M-15,_ *bla*_CMY-16,_ *bla*_OXA-1_, *bla*_OXA-10_, *bla*_TEM-1D,_ *bla*_SHV-11_ | 0 | *mrkD, fimH* |
| Kleb_106 | *bla*_NDM-1_, *bla*_SHV-187_ | 0 | *mrkD, fimH* |
| Kleb_107 | *bla*_NDM-1_, *bla*_SHV-187_ | 0 | *mrkD, fimH* |
| Kleb_108 | *bla*_NDM-1_, *bla*_OXA-48_, *bla*_CTX-M-15,_ *bla*_SHV-11_ | 0 | *-* |
| Kleb_109 | *bla*_NDM-1,_ *bla*_SHV-11_ | 0 | *ybtA, mrkD, fimH* |
| Kleb_110 | *bla*_NDM-1_, *bla*_OXA-48_, *bla*_CTX-M-15,_ *bla*_SHV-11_ | 0 | *mrkD, fimH* |
| Kleb_EMO_1 | *bla*_CTX-M-15_ | 0 | *mrkD, fimH* |
| Kleb_EMO_2 | *bla*_KPC-3_ | 0 | *mrkD, fimH* |
| Kleb_EMO_3 | *bla*_OXA-48_ | 1 | *ybtA, ybtS, mrkD, fimH* |
| Kleb_EMO_4 | *-* | 0 | *mrkD, fimH* |
| Kleb_EMO_5 | *-* | 0 | *-* |
| Kleb_EMO_6 | *bla*_KPC-3_ | 1 | *ybtA, ybtS, mrkD, fimH* |
| Kleb_EMO_7 | *bla*_SHV-52_*, bla*_PC1-6_ | 0 | *-* |
| Kleb_EMO_8 | *bla*_SHV-52_ | 0 | *rmpa, mrkD, fimH* |
| Kleb_EMO_9 | *bla*_NDM-1_*, bla*_OXA-48_ | 1 | *ybtS, mrkD, fimH* |

^1^Virulence scores according to KLEBORATE online tool (available at <https://github.com/klebgenomics/Kleborate>, accessed on 23^rd^ March 2026): 0: no virulence genes; 1: *ybt*; 2: *ybt*, *clb*; 3: *iuc*; 4: *ybt*, *iuc*; 5: *ybt*, *clb*, *iuc.* Virulence genes detected by Real-Time PCR.
